# One-week sleep hygiene education improves episodic memory in young but not in older adults during social isolation

**DOI:** 10.3389/fpsyg.2023.1155776

**Published:** 2023-08-01

**Authors:** Leonela Magali Tassone, Malen Daiana Moyano, Fernando Laiño, Luis Ignacio Brusco, Rodrigo Ezequiel Ramele, Cecilia Forcato

**Affiliations:** ^1^Laboratorio de Sueño y Memoria, Departamento de Ciencias de la Vida, Instituto Tecnológico de Buenos Aires (ITBA), Buenos Aires, Argentina; ^2^Consejo Nacional de Investigaciones Científicas y Tecnológicas (CONICET), Buenos Aires, Argentina; ^3^Fundación Instituto Superior de Ciencias de la Salud, Buenos Aires, Argentina; ^4^CENECON, Centro de Neuropsiquiatría y Neurología de la Conducta (CENECON), Buenos Aires, Argentina; ^5^Centro de Inteligencia Computacional, Instituto Tecnológico de Buenos Aires (ITBA), Buenos Aires, Argentina

**Keywords:** episodic memory, sleep hygiene, social isolation, young adults, older adults

## Abstract

Memory formation is a dynamic process that comprises different phases, such as encoding, consolidation and retrieval. It could be altered by several factors such as sleep quality, anxiety, and depression levels. In the last years, due to COVID-19 pandemic, there was a reduction in sleep quality, an increase in anxiety and depressive symptoms as well as an impairment in emotional episodic memory encoding, especially in young adults. Taking into account the profound impact of sleep quality in daily life a series of rules has been developed that are conducive to consistently achieving good sleep, known as sleep hygiene education. These interventions have been shown to be effective in improving sleep quality and duration and reducing depressive and anxiety symptoms. Here we propose the implementation of a brief sleep hygiene education to improve sleep quality and memory performance as well as to diminish anxiety and depressive scores. For that, participants were divided into two groups: Sleep hygiene education and control group. After that, they were evaluated for anxiety, depression, and sleep quality levels and trained on an episodic memory task. They were tested immediately after (short-term test) and also 1 week later (long-term test). This procedure was also performed before the sleep hygiene education and was taken as baseline level. We found that episodic memory performance for young adults improved for the SHE group after intervention but not for older adults, and no improvements in emotional variables were observed. Despite not observing a significant effect of the intervention for young and older adults regarding the sleep quality scores, we consider that there may be an improvement in sleep physiology that is not subjectively perceived, but would also have a positive impact on memory processes. These results show that even a sleep hygiene education of 1 week could improve cognition in young adults when acute memory and sleep impairment occurs, in this case, due to the isolation by COVID-19 pandemic. However, we suggest that longer interventions should be implemented for older adults who already experience a natural decline in cognitive processes such as episodic memory formation.

## Introduction

Memory formation is a dynamic process that comprises different phases, such as encoding, consolidation and retrieval (Dudai, 2002). During encoding, stimuli are perceived, resulting in a neuronal representation that generates a new memory trace. This memory trace is initially labile followed by a process of stabilization and integration known as consolidation. Finally, during retrieval, the stored information can be recalled (Dudai et al., 2015).

Sleep is a natural, reversible, homeostatically regulated physiological state of rest, conserved across evolution (Vorster and Born, 2014). It involves low levels of physiological activity and a reduction in the response to external stimuli (Eugene and Masiak, 2015). Sleep plays a fundamental role in the memory process. It has been shown that recently acquired information is reactivated during sleep, promoting the transfer and redistribution of the information from the hippocampus to the neocortex favoring memory consolidation (Rasch and Born, 2013). Moreover, sleep facilitates memory integration and reorganization (Conte and Ficca, 2013). In fact, new acquired memories are not only made permanent and durable through consolidation, but are also associated with and reorganized with older memories.

Furthermore, during wakefulness, the constant encoding of information leads to a net synaptic strength increase in the brain (Tononi and Cirelli, 2014). This increase boosts cellular energy requirements, saturating learning processes. Thus, subsequent sleep serves to globally downscale synaptic strength reestablishing cellular homeostasis leaving synapses available for future encoding (Diekelmann and Born, 2010; Tononi and Cirelli, 2014).

Sleep deprivation or disruption causes severe cognitive damage and emotional problems (Walker, 2009; Medic et al., 2017). Numerous studies showed that diminishing total sleep time as well as sleep disruptions, resulted in negative effects on several neurocognitive functions such as episodic memory, working memory and psychomotor vigilance tasks. It further increases errors of omission (lapses) and commission (wrong responses), and impairs memory acquisition (Dinges et al., 1997; Belenky et al., 2003; Van Dongen et al., 2003; Goel et al., 2009; Yeh et al., 2018, 2021; Hokett et al., 2021).

Regarding normal aging, it involves deficits in encoding, consolidating, and recalling information (Grady and Craik, 2000). In addition, one of the most common subjective memory complaints in older adults is related to the difficulty in acquiring and remembering new information (Craik and Rose, 2012), being episodic memories the most affected (Light, 1991; Craik and Jennings, 1992). Furthermore, normal aging is accompanied by changes in sleep structure, including both a decrease in total sleep time and in sleep efficiency as well as increase in sleep fragmentation (Ohayon et al., 2004; Pace-Schott and Spencer, 2011; Li et al., 2018).

Furthermore, sleep plays an important role in mood regulation. It has been shown that poor sleep quality and inadequate sleep parameters leads to increased anxiety symptoms and psychological stress (Manzar et al., 2021). Furthermore, in a meta-analysis conducted by Scott et al. (2021), it was found that interventions oriented to improve sleep quality lead to a significant effect on diminishing depression symptoms, anxiety and rumination (Scott et al., 2021).

In the last years, due to Covid-19 pandemic, there was an increase in anxiety and depressive symptoms as well as a reduction of the sleep quality throughout the population, especially in young adults (Sher, 2020; Solomou and Constantinidou, 2020; Etchevers et al., 2021; Nelson and Bergeman, 2021; Varma et al., 2021; Bonilla et al., 2022; Leon et al., 2022). Moreover, regarding sleep quality and quarantine effects, it was found that people who experienced reduced sleep time, and those who had delayed sleep time, had clinically important worsening on measures of stress and anxiety (Robillard et al., 2020). Moreover, the context of the pandemic directly impacted memory processes. It was found that encoding of episodic emotional content was impaired in young adults, and that the higher levels of anxiety worsened the encoding of emotional episodic information (Leon et al., 2022).

Taking into account the deep impact of sleep quality in daily life there is a series of rules that has been developed that are conducive to sleeping well on a regular basis, known as sleep hygiene (Chen et al., 2010). This intervention refers to a set of practices and environmental factors that are related to a good sleep quality and the effects that some habits and substances that are ingested before bedtime have on the homeostatic conduction of sleep and the circadian cycle (Yang et al., 2010). In relation to some of the recommendations established for people with daytime work and without rotative schedules, we can enumerate the following: (a) limit exposure to bright light after sunset to avoid inhibiting melatonin secretion, which is the main sleep-inducing hormone synthesized in the dark (Cardinali, 2007; Zisapel, 2018). (b) Perform physical exercise: in a meta-analysis conducted by Kubitz et al. (1996), it was found that both sporadic and regular physical exercise increases deep sleep and decreases sleep onset latency. However, it is recommended to avoid strenuous exercise within 4 h before going to sleep, as it increases sleep onset latency (Youngstedt et al., 1997). (c) It is also recommended taking short naps, lasting no more than 30 min, between 1:00 p.m. and 3:00 p.m. This enhances alertness levels and improves cognitive performance, without causing significant sleep inertia or impairing the propensity to sleep at night (Jefferson et al., 2005; Lovato and Lack, 2010). (d) In relation to food and eating habits, it is recommended to avoid consuming stimulating drinks, such as coffee, within 4–6 h before going to bed (Cheek et al., 2004). This is because caffeine is a potent adenosine receptor antagonist, which reduces sleep efficiency, shortens total sleep time, increases sleep latency, and decreases the percentage of slow wave sleep (Stepanski and Wyatt, 2003; Drapeau et al., 2006; Ogeil and Phillips, 2015). Regarding diet, it is recommended to avoid the consumption of foods with diuretic properties or those that are difficult to digest, as well as foods rich in tyrosine (such as meat and sausages), as they are precursors of dopamine, a neurotransmitter involved in the wakefulness system (Hase et al., 2015). On the other hand, it is recommended to include foods rich in tryptophan in dinner, as it is a precursor of melatonin (Hajak et al., 1991), as to consume carbohydrates, as they increase insulin secretion and improve the bioavailability of tryptophan (Silber and Schmitt, 2010). Additionally, it is recommended to consume foods rich in Omega-3 fatty acids, magnesium, calcium, and Vitamin B is recommended, as they are necessary for the conversion of serotonin to melatonin (Richardson, 2015). (e) Furthermore, current recommendations emphasize the importance of reducing mental activity at least 30 min before going to sleep, as ruminating on problems and worries in bed raises alertness, impairing relaxation and subsequent sleep onset, with excessive mental activity correlating to a propensity for insomnia (Harvey, 2000).

Sleep hygiene education has been shown to be effective in improving sleep quality sleep duration, reducing depressive symptoms, anxiety and improving sleep (Blunden, 2014; Hershner and O'Brien, 2018; Sharma and Srivastava, 2018; Lemrasky et al., 2019), as well as in reducing insomnia (Blunden, 2014). Sleep hygiene is an economic tool, easily available and simple to manage for those who need to improve their sleep quality (Ju and Woo, 2016).

Here we proposed the sleep hygiene education to be quickly implemented in young and older adults, during periods of social isolation such as the one we had recently experienced during the COVID-19 pandemic, to improve the sleep quality positively impacting on memory processes and emotional variables such as anxiety and depressive symptoms.

## Materials and methods

The study was conducted online during the COVID-19 quarantine and was performed on 4 days with a one-week interval between each day. We used Google Meet Platform to share the online videos. The protocol and the informed consent were approved by the Alberto C. Taquini Institute Biomedical Research Ethics Committee in accordance with the principles expressed in the Declaration of Helsinki. The study was carried out within the first period of the Preventive and Mandatory Social Isolation (ASPO) by COVID-19 in Argentina.

### Participants

Thirty-nine young adults (27 females and 12 males, ages ranged between 20–40 years: M = 29.3, SEM = 1.0) and thirty-nine healthy older adults (31 females and 8 males, ages ranged between 60–85 years: M = 67,4, SEM = 1.0) volunteered for the study. Data from seven adults were excluded from the analysis for the following reasons: did not finish the study (3) and had higher BDI scores than the cut-off point of 21 (4). They were recruited *via* social media platforms from our laboratory (Twitter, Facebook, and Instagram).

The final sample consisted of thirty-four young adults (22 females and 12 males) and thirty- seven older adults (29 females and 8 males). Young adults were graduate and undergraduate students from Argentina, with a mean of 15.1, SEM = 0.4 years of education and their ages ranged between 20–40 years (M = 29.1, SEM = 1.1). They had no history of neuropsychiatric disorders, and they did not use drugs. Older adults’ ages ranged between 60–85 years (M = 67.3 SEM = 1.1). They had completed secondary school, with a mean of 15.0, SEM = 0.3 years of education and had no history of previous neurological or psychiatric disorders.

Prior to being enrolled in the experiment, subjects completed psychological and neurocognitive assessment, including State–Trait Anxiety Inventory (STAI) (Spielberger et al., 1983), Beck Depression Inventory II (BDI- II) (Beck et al., 1996), Morningness-Eveningness Questionnaire (MEQ) (Horne and Ostberg, 1976) and Pittsburgh Sleep Quality Index (PSQI) (Buysse et al., 1989) (Table 1). Older adults also completed the Signoret Mnesic efficiency battery (BEM-144) (Signoret, 1991; Leis et al., 2018) (Table 2).

**Table 1:** Psychological measures and sleep quality index.

Groups	MEQ	BDI	BDI	STAI State	STAI State	PSQI	PSQI
		Day 1	Day 22	Day 1	Day 22	Day 1	Day 22
Young adults-SHE	47.22 ± 2.18	11.17 ± 1.28	7.89 ± 1.33	41.17 ± 2.05	40.11 ± 2.30	4.72 ± 0.60	4.72 ± 0.56
Young adults-CTL	47.19 ± 2.23	10.94 ± 1.32	9.31 ± 1.55	40.50 ± 2.26	38.25 ± 2.01	6.25 ± 0.67	5.38 ± 0.72
Older adults-SHE	54.25 ± 2.17	8.80 ± 1.10	7.95 ± 1.08	38.40 ± 1.69	38.45 ± 1.89	8.55 ± 0.89	6.80 ± 0.74
Older adults- CTL	57.88 ± 2.86	6.35 ± 1.05	5.76 ± 1.31	34.59 ± 1.27	33.65 ± 1.76	5.94 ± 1.02	5.71 ± 1.04

Mean State–Trait Anxiety Inventory (STAI), Beck Depression Inventory II (BDI II), Pittsburgh Sleep Quality Index (PSQI) and Morningness-Eveningness Questionnaire (MEQ) ± SEM on day 1 (baseline) and day 22 (after sleep hygiene or control intervention). SHE stands for the sleep hygiene education group, CTL stands for the control group.

**Table 2:** Neurocognitive assessment.

Groups	Immediate	Delayed	Serial	Serial	Recognition
	Recall	Recall	Learning	Recall	
Older adults-SHE	10.60 ± 0.27	9.85 ± 0.33	9.05 ± 0.38	7.60 ± 0.37	10.45 ± 0.32
Older adults-CTL	11.18 ± 0.18	10.00 ± 0.30	9.18 ± 0.21	8.59 ± 0.30	10.88 ± 0.15
*p- value*	0.09	0.74	0.55	0.10	0.25

Mean scores at Signoret Mnesic efficiency battery (BEM-144), ± SEM for older adults. SHE stands for the sleep hygiene education group, CTL stands for the control group.

Data from seven adults were excluded from the analysis for the following reasons: did not finish the study (3) and had higher BDI scores than the cutoff point of 21 (4). Among the participants that concluded the experiment, three gift cards from a bookstore were raffled.

### Procedure

Each session was carried out between 10 a.m. and 4 p.m. On day 1, participants signed the online consent form to participate in the study and received a link *via* email to complete a sociodemographic questionnaire, psychological measures, and sleep quality assessment: Beck Depression Inventory-II (BDI II) State–Trait Anxiety Inventory (STAI), the Pittsburgh Sleep Quality Index (PSQI) and the Morningness–Eveningness Questionnaire (MEQ). After the online form was completed, they received a link for the video call. Participants were asked to turn the volume up to maximum and pay attention to the screen. Immediately after the experimenter’s screen was shared, a 15 s demo video was played to check audio and internet connection. Participants were advised that if they had any problem during the video presentation, they should notify the experimenter. Immediately after, the episodic memory video was presented. After that, participants performed the short-term free recall. One week later, participants entered a video call link previously sent *via* email. On that day, they performed the long-term free recall, the memory recognition, and the temporal episodic order task. After the session, participants received the intervention. The sleep hygiene education group received instructions to perform the sleep hygiene activities. The control group did not receive any instructions and continued with their usual routines. During the following 2 weeks, all participants had to complete an online form with the activities carried out. The procedure was repeated in the same way 1 week later, alternating the stories among the participants. The entire experiment was conducted and supervised by one experimenter.

## Session 1 and 3

They were formed by the episodic memory training (story 1 or 2) and the short-term free recall testing (story 1 or 2).

### Episodic memory training

The study was carried out using 2 videos with different episodic memory stories formed by neutral emotional content. Both were composed of a 3-min audio video consisting of 11 consecutive images, each of them was presented on the screen for 15 s while an auditory narrative was being played describing the story. Each story was about a person’s daily routine.

### Short-term free recall testing

After watching the video, subjects had to report aloud everything they remembered about what they had seen and heard in the video, in as much detail as possible. All oral reports were recorded, and the number of correct reported details about the actions, people, objects, and elements of the environment provided were counted as the subject’s memory performance. To decide if the detail was correct or false, the story provided by the subject was compared with the original oral story of the video. Each detail or its synonym was counted only once, regardless how many times it was repeated in the free recall. The instruction was “Now I am going to ask you to describe all you have seen and heard in the video in as much detail as possible.”

## Session 2 and 4

It was formed by the long-term free recall testing, the memory recognition, and the episodic order tasks (story 1 or 2).

### Long-term free recall testing

Participants were asked to orally recall the content of the video watched 1 week before. Subjects had to report aloud everything they remembered about what they had seen and heard in the video, in as much detail as possible. Analysis of correct answers was carried out in the same way as for the short-term free recall testing.

### Memory recognition

The memory recognition task consisted of 11 images that were presented altogether, in which 5 of them were already presented in the video of the training session and 6 were new. These 6 new photos were similar in content to the ones shown in the story. The images were listed from letter A to K. Participants had to choose which ones they considered to have seen in the video. Their answers were registered and were offline scored by experimenters.

### Temporal episodic order task

After the recognition task, subjects were asked to order the chosen images temporarily, according to the episodic order they remembered. Their answers were registered and were offline scored by experimenters.

### Sleep hygiene education

Participants in the sleep hygiene education group received specific and individual instructions to perform the activities (S2 Appendix). All participants received an explanatory video in which all the activities they had to carry out according to their chronotype were detailed. The video included explanations about routine times to wake up and go to bed, recommended or to avoid drink and food intake, limited daytime napping, recommended times for physical activity, sun exposure, modifying the environment (e.g., reduce impact of noise/light), and avoidance of the use of electronic light-emitting devices before bedtime. After watching the video, participants received an online form in which they had to complete daily activities that they performed (S1 Data).

## Tests and questionnaires

### Sociodemographic questionnaires

This questionnaire consisted of answering questions about age, years of education and gender.

### Sleep quality and psychological measures

We evaluated the quality of sleep using the Pittsburgh Sleep Quality Index (PSQI) the psychological measures using Beck Depression Inventory II (BDI-II) and State–Trait Anxiety Inventory (STAI). Furthermore, we evaluated the chronotype using Morningness-Eveningness Questionnaire (MEQ) to give the sleep hygiene education considering their chronotypes.

### Neurocognitive assessment

Older adults also completed a brief neurocognitive screening before beginning participation in the study to evaluate general cognitive functioning, using the Signoret Mnesic efficiency battery (BEM-144).

### Experimental groups

Young and older adults were randomly assigned to one of two conditions: “Sleep hygiene education group” (SHE) or “Control group” (CTL).

*Sleep hygiene education* (SHE) *group.* Participants were first trained on an episodic memory task on day 1 and immediately tested (Session 1). One week later (day 8- Session 2) they performed the long-term free recall testing, the memory recognition, and the episodic order task. After that, they received the sleep hygiene education they have to carry out the next two weeks. One week later (Session 3 – day 15), they were trained and immediately tested on a new episodic memory task. The following week (Session 4 – day 22), they performed the free recall long term testing, the memory recognition, and the episodic order task.

*Control* (CTL) *group*: Participants were first trained on an episodic memory task on day 1 and immediately tested (Session 1). One week later (day 8– Session 2), they performed the long-term free recall testing, the memory recognition, and the episodic order task. After that, they continued with their normal sleep routines. One week later (Session 3 - day 15), they were trained and immediately tested on a new episodic memory task. The following week (Session 4 – day 22), they performed the free recall long term testing, the memory recognition, and the episodic order task.

### Statistical analysis

Statistical analysis was performed with SPSS version 25 (IBM Corporation). We calculated the percentage of correct responses reached in each training and testing free recall session. As each story had a different total of possible correct details to be remembered (Story 1: 125, Story 2: 122), we normalized the correct recalled details using the percentage of correct details (i.e., number of correct details/possible correct details*100). We first analyzed the percentage of correct responses at short-term and long-term testing 1 (baseline level) with a repeated measures ANOVA with “group” as inter-subject factor with two levels (“SHE” and “CTL”) and “time” as a repeated measure with 2 levels (short-term and long-term evaluation) for young and older adults. We also analyzed the percentage of correct responses at short-term and long-term testing 2 (after intervention) with a repeated measures ANOVA with “group” as inter-subject factors with 2 levels (“SHE” and “CTL”) and “time” as a repeated measure with 2 levels (short-term and long-term evaluation).

In relation to the memory recognition task, both the number of correct and wrong choices were analyzed. Regarding the temporal episodic order task, the number of deviations with respect to the correct temporal order was counted. Deviations were accounted by comparing the chosen images with the correct episodic order of the story. If the participants chose a wrong photo but placed the event in the correct place in the episodic order, it was taken as valid, since in this task only the episodic order of events was evaluated. For this analysis we performed a one-way ANOVA, with groups as between subject’s factor with two levels (SHE and CTL).

We analyzed the scores obtained at BDI, STAI and PSQI on day 1 with separately two way ANOVAs with “group” as between subjects’ factor with two levels (control-sleep hygiene) and “age” as between subjects factor with two levels (young, older). Followed by simple effects analyses in case of significant interaction. We further analyzed the State Anxiety, Trait Anxiety, BDI-II, PSQI and MEQ in both groups with repeated measures ANOVA with 2 levels (day 1- baseline level and day 22- final level).

In relation to sleep hygiene activities carried out during the participation in the study, we performed a one-way ANOVA, with groups as between subject’s factor with two levels (sleep hygiene education - control).

In order to examine if memory processes could be affected by other variables such as emotional variables, sleep quality index or sleep hygiene activities, we further analyzed STAI, BDI II, PSQI and sleep hygiene activities and memory performances in the two conditions (SHE and CTL) in young and older adults with Pearson correlations. Alpha was set at 0.05.

## Results

**Figure 1:**
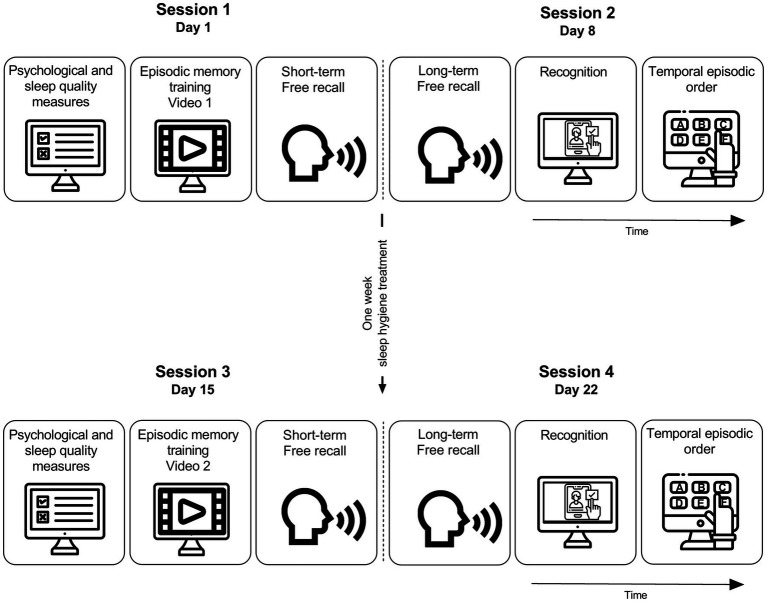
Experimental procedure. The procedure was divided into four sessions. On day 1 participants completed the psychological and sleep quality measures. Then, they watched the video and finally made a free recall of it (short-term testing). On session 2 (1 week later, day 8) participants had to freely recall the video watched on session 1 (long-term testing). After that, participants had to recognize the correct images (Recognition task) and then had to order the chosen images (Temporal episodic order). After session 2 participants were divided into two groups, one went through the sleep hygiene education (SHE), and the other continued with their normal sleep routines (CTL). On session 3 (1 week later, day 15) all groups completed the psychological and sleep quality measures. Then, they watched the video and finally made a free recall of it (short-term testing). On session 4 (1 week later, day 22) participants had to freely recall the video watched on session 3 (long-term testing). After that, participants had to recognize the correct images (Recognition task) and then had to order the chosen images (Temporal episodic order). Icons taken from Freepik [https://www.flaticon.com/authors/freepik].

In order to study the effect of one week of sleep hygiene education in young and older adults, we performed a 22-day study divided into 4 sessions (Figure 1). The first two sessions corresponded to the baseline levels. On session 1 (day 1), participants were trained in an episodic memory task and immediately tested. On session 2 (day 8), participants were tested. After that, half of the participants received the sleep hygiene education (SHE) for one week and the other half continued with their normal sleep routines (CTL). The sleep hygiene education group received instructions to begin the sleep hygiene activities and the control group continued with their usual sleep routines. After one week, on session 3 (day 15), all participants were trained in a new episodic memory task, and then were tested on day 22 (session 4).

### Memory variables

#### Free recall

For both young and older adults, the SHE groups and the CLT groups had the same baseline levels for the percentage of correct responses at short and long-term evaluation (young adults short-term evaluation, SHE group: 53.49 ± 2.45; CTL: 46.54 ± 2.41; long-term evaluation, SHE group: 39.41 ± 2.88; CTL: 32.80 ± 3.29; F_group_(1,32) = 3.70, *p* = 0.06. Older adults short-term evaluation, SHE group: 48.72 ± 2.48; CTL: 48.15 ± 2.49; long-term evaluation, SHE group: 31.40 ± 3.65; CTL: 25.18 ± 1.50; F_group_(1,35) = 1.05, *p* = 0.31). Furthermore, for both young and older adults, there was a significant decay in the percentage of correct responses between the short and long-term testing independent of the group (young adults, F_time_(1,32) = 63.87, *p* < 0.001, η^2^_p_ = 0.66 and F_group*time_(1,32) < 0.001, *p* = 0.92; older adults, F_time_(1,35) = 98.00, p < 0.001, η^2^_p_ = 0.73 and F_group*time_(1,35) = 1.92, *p* = 0.17).

**Figure 2:**
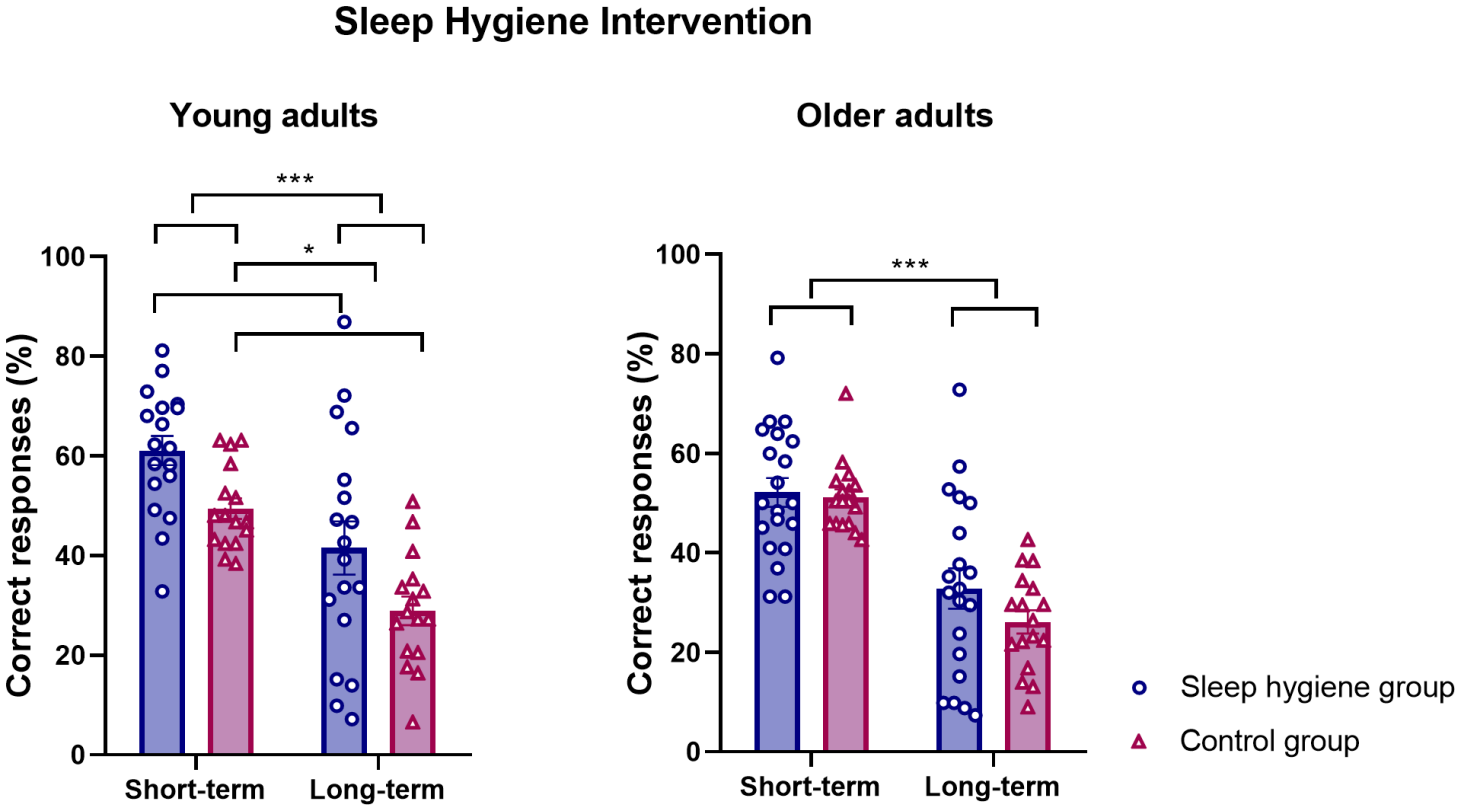
Sleep hygiene intervention effect on memory performance. Mean percentage of correct responses at short and long-term free recall after the sleep hygiene education ± SEM for young and older adults.

After the intervention, the SHE group performed significantly better than the control group for young adults (Figure 2. short-term evaluation, SHE group: 61.06 ± 2.93; CTL: 49.45 ± 2.08; long-term evaluation, SHE group: 41.53 ± 5.35; CTL: 28.91 ± 2.85, F_group_(1,32) = 7.48, *p* = 0.01, η^2^_p_ = 0.19) independently of the time of evaluation (F_group*time_(1,32) = 0.03, *p* = 0.84). Furthermore, there was a significant decay on the percentage of correct responses between the short and long-term testing due to natural forgetting (F_time_(1,32) = 58.20, *p* < 0.001, η^2^_p_ = 0.64). However, no significant effect of the hygiene education was observed for older adults (Figure 2. Short-term evaluation, SHE group: 52.14 ± 2.88; CTL: 51,13 ± 1,68; long-term evaluation, SHE group: 32.82 ± 4.06; CTL: 26.12 ± 2.33, F_group_(1,35) = 1.15, *p* = 0.29), but there was a significant decay between the short and long-term testing independent of the group as in younger adults (F_time_(1.35) = 94.0, *p* < 0.001, η^2^_p_ = 0.73), F_group*time_(1,35) = 1.5, *p* = 0.22).

#### Recognition

The SHE and CTL groups had the same baseline levels for the number of correct chosen images at the first recognition task (Table 3, young adults: *F*(1,32) = 0.60, *p* = 0.44, older adults: *F*(1,35) = 0.33, *p* = 0.56) as well as for the number of wrong chosen images (Table 3, young adults: F(1,32) =0.13, *p* = 0.71; older adults F(1,35) =0.56, *p* = 0.45).

**Table 3:** Image recognition and temporal episodic order.

Groups	Correct image recognition	Correct image recognition	False image recognition	False image recognition	Temporal order deviation	Temporal order deviation
	Day 1	Day 22	Day 1	Day 22	Day 1	Day 22
Young adults-SHE	4.33 ± 0.18	4.11 ± 0.20	1.00 ± 0.23	0.72 ± 0.16	0.22 ± 0.13	0.44 ± 0.18
Young adults-CTL	4.06 ± 0.31	3.56 ± 0.26	0.88 ± 0.26	1.00 ± 0.24	0.50 ± 0.22	0.81 ± 0.25
Older adults-SHE	3.35 ± 0.32	3.80 ± 0.27	1.85 ± 0.33	3.35 ± 0.32	1.00 ± 1.05	1.05 ± 0.25
Older adults- CTL	3.59 ± 0.24	3.71 ± 0.27	1.53 ± 0.26	3.59 ± 0.24	0.53 ± 0.15	0.71 ± 0.19

Mean scores at correct and false Image recognition and deviations of temporal episodic order ± SEM on day 1 (baseline) and day 22 (after Sleep Hygiene education or control). SHE stands for the sleep hygiene education group, CTL stands for the control group.

Contrary to our expectations, there were no positive effect of the hygiene education for recognition (Table 3, young adults correct images: F(1,32) = 2.94, *p* = 0.09; wrong images: F(1,32) = 0.96, *p* = 0.33; older adults correct images: F(1,35) = 0.06, *p* = 0.80; wrong images: F(1,35) = 0.33, *p* = 0.56).

#### Temporal episodic order

There were no significant differences on deviations between groups neither for young nor older adults at baseline level (Table 3, young adults’ F(1,32) = 1.22, *p* = 0.27; older adults’ F(1,35) = 2.52, *p* = 0.12). Furthermore, contrary to our hypothesis, there were no significant differences between groups neither for young nor for older adults (Table 3, young adults’ F(1,32) = 1.47, *p* = 0.23; older adults’ F(1,35) = 1.17, *p* = 0.28).

#### Sleep quality and psychological measures

Young adults showed significantly more BDI score at day 1 than older adults [F_age_(1,67) = 8.05, *p* < 0.05, η^2^_p_ = 0.10], but no differences were found between groups [F_group_(1,67) = 0.23, *p* = 0.63] and no age*group interaction [F_age*group_(1,67) = 0.38 *p* = 0.53]. In relation to anxiety scores, young adults obtained higher scores in the STAI state test [F_age_(1,67) = 5.50, *p* < 0.05, η^2^_p_ = 0.07], but no differences were found neither between groups [F_group_(1,67) = 1.46, *p* = 0.23] nor age*group interaction [F_age*group_(1,67) = 0.72 *p* = 0.39].

Regarding PSQI on day 1, there was a significant age*group interaction [F_age*group_(1,67) = 6.25 *p* < 0.05, η^2^_p_ = 0.08]. Thus, we performed simple effects analyses of “group” within each level of “age.” For the young adults, there was no significant difference between the sleep hygiene treatment and control group [*F*(1,67) = 1.63, *p* = 0.20]. However, for the older adults, the sleep hygiene education group has higher scores than the control group at day 1 [F(1,67) = 5.18, *p* = *p* < 0.05, η^2^_p_ = 0.07]. We further performed simple effects analyses of “age” within each level of “group.” For the control condition, we found that there were no significant differences between young and older adults [F(1,67) = 0.06, *p* = 0.79], but for the sleep hygiene condition, older adults had higher PSQI scores [F(1,67) = 11.50, *p* < 0.005, η^2^_p_ = 0.14].

Contrary to our hypotheses, we observed no significant effect of the intervention for young and older adults regarding the sleep quality (Table 1, young adults: F_group_(1,31) = 1.75, *p* = 0.19; F_time_(1,32) = 1.35, *p* = 0.25, F_group*time_(1,32) = 1.35, p = 0.25; older adults: F_group_(1,35) = 2.36, *p* = 0.13; F_time_(1,35) = 4.05, *p* = 0.05, F_group*time_(1,35) = 2.35, p = 0.13), the level of anxiety (Table 1, young adults: F_group_(1,32) = 0.20, *p* = 0.65; F_time_(1,32) = 1.93, *p* = 0.17, F_group*time_(1,32) = 0.25, *p* = 0.61; older adults: F_group_(1,35) = 3.68, *p* = 0.06; F_time_(1,35) = 0.26, p = 0.61, F_group*time_(1,35) = 0.32 *p* = 0.57). For young adults, regarding the level of depression we found a significant decay in the BDI II score between baseline and after sleep hygiene education, independently of the group (Table 1, young adults: F_time_(1,32) = 16.56, *p* < 0.001, η^2^_p_ = 0.34). However, for older adults there was no significant differences in the BDI II score between baseline and after sleep hygiene education (F_time_(1,35) = 1.86, *p* = 0.18) Furthermore, no significant effect of the hygiene education was observed neither for young nor for older adults (Table 1, young adults: F_group_(1,32) = 0.10, *p* = 0.74; older adults: F_group_(1,35) = 2.30, p = 0.13) and no significant interaction (Table 1, young adults: F_group*time_(1,32) = 1.88, p = 0.18, older adults: F_group*time_(1,35) = 0.06, *p* = 0.80).

In relation to the sleep hygiene activities carried out during the study, there was a greater amount of sleep hygiene activities in the sleep hygiene education group for both young adults [*F*(1,26) = 63.55, p < 0.005, η^2^_p_ = 0.71. SHE: M = 26.9 SEM = 1.2 CTL: M = 9.36 SEM = 1.8] and for older adults [*F*(1,33) = 107.11, p < 0.005, η^2^_p_ = 0.76. SHE:M = 33.0, SEM = 1.5, CTL: M 10.25, SEM = 1.5].

### Exploratory correlation analyses

Furthermore, we analyzed the correlations between the values obtained in the psychological measures, sleep quality assessment and sleep hygiene activities and the memory performance.

For the young adult’s hygiene group, we found significant negative correlations between percentage of correct details on free recall on day 15 and STAI state score (*r* = −0.57, *p* = 0.01) and BDI score (*r* = −0.50, *p* = 0.03). For the young adult’s control group, we found significant negative correlations between percentage of correct details on free recall on day 15 and STAI state score (*r* = −0.53, p = 0.03) and BDI score (*r* = −0.63, *p* < 0.01). For older adults in the control group, we found a moderate significant negative correlation between percentage of correct details on free recall on day 22 and total BDI score (*r* = −0.53, *p* = 0.02). No other significant correlations were found (−0.01 < *r* < 0.4, all ps > 0.06).

## Discussion

In the present study, we showed that a short sleep hygiene intervention of one-week improved memory encoding and consolidation in young adults, but not in older adults. We attribute these differences to the differential impact of social isolation on these two age groups. On one hand, young adults’ routines and lifestyle were suddenly altered as a result of the quarantine showing greater scores on anxiety and depression levels (Czeisler et al., 2020). On the other hand, in older adults the proactive coping might have functioned as a resilience factor (Czeisler et al., 2020; Nelson and Bergeman, 2021; Pearman et al., 2021). In this line, many studies have shown that young adults were the most affected in relation to emotional symptoms. Varma et al. (2021) found that young people had more stress, anxiety and depression scores compared to older adults groups during COVID-19 pandemic. Similarly, Solomou and Constantinidou (2020) found an increase of COVID-19 related anxiety and depression symptoms, especially in young adults between 18 to 30 years, who were the most affected with mental symptoms.

Furthermore, it has been widely demonstrated that emotional variables could negatively impact cognition (Kizilbash et al., 2002; Bolton and Robinson, 2017). Depressive symptoms affect different cognitive domains, such as executive functions, speed processing and episodic memory (Watts et al., 1987; McDermott and Ebmeier, 2009; Rock et al., 2014). Regarding anxiety, low anxiety symptoms have been associated with better cognitive performances, while severe anxiety symptoms were negatively associated assuming a curvilinear relationship between anxiety and cognition, that could modulate memory processes (Bierman et al., 2005).

In relation to that, in the present study we found a significant negative correlation between anxiety and depression scores and the percentage of correct responses on free recall for both groups. These correlations are in line with previous studies showing that the higher scores at anxiety and depression tests, the worse memory performance (Airaksinen et al., 2005; Bolton and Robinson, 2017; Leon et al., 2022). In line with this, the free recall process is usually mostly affected than recognition in what is included the so-called memory cues. It is widely accepted that recognition can be achieved either by familiarity or recollection process, while free recall depends entirely on conscious recollection (Mandler, 1980), since there are no other processes like familiarity available to lean on and help memory retrieval (Dobbins et al., 1998). Moreover, there were studies that have drawn attention to the probability of ceiling effects on many recognition tasks performed by young adults (Uttl et al., 2007; Danckert and Craik, 2013). In line with that, we consider that changes on free recall memory performances are more susceptible to intervention.

In relation to sleep habits improvements, although the baseline measurements of habits prior to sleep hygiene education were not taken, we found a higher number of habits related to good sleep hygiene, showing a significant difference between the group that received the sleep hygiene education and control group. These results show that there was indeed a difference in the habits that promote a good sleep quality in the group that underwent sleep hygiene education, as opposed to the control group. This objective difference could be producing a change in the sleep physiology, which therefore has a positive impact on the memory performance of young adults.

Taking all together, we suggest that sleep hygiene education had a positive impact on memory processes only for young adults. This group was particularly affected by the pandemic context, and therefore, an improvement on sleep habits could have a positive outcome on memory performance. It is important to note, however, that one limitation of our study was the lack of pre-existing anxiety and depression scores to examine the increase of these variables in response to the pandemic context. Nevertheless, we did observe significant differences in anxiety and depression scores between young and older adults, with young adults obtaining the highest scores. These findings are in line with previous studies indicating that young adults were more affected by the Covid-19 isolation measures, resulting in higher levels of anxiety and depression compared to older adults (Sher, 2020; Solomou and Constantinidou, 2020; Etchevers et al., 2021; Nelson and Bergeman, 2021; Varma et al., 2021; Bonilla et al., 2022; Leon et al., 2022). Regarding PSQI scores, while older adults had higher scores on day 1, this could be attributed to normal changes in sleep patterns associated with aging (Ohayon et al., 2004; Li et al., 2018; Pace-Schott and Spencer, 2011). Older adults may have a more negative subjective perception of their sleep quality compared to younger adults, as reflected in their PSQI scores. Changes in sleep and subjective complaints among older adults have been extensively documented in previous studies (Foley et al., 1999; Miner and Kryger, 2017). However, in the case of young adults without sleep disorders, such changes would not be expected. Given the design of our study and abrupt nature of the quarantine, we were unable to collect measures prior to the isolation period. Nonetheless, considering the consistent findings of increased anxiety and depression scores in numerous studies (Robillard et al., 2020; Sher, 2020; Solomou and Constantinidou, 2020; Etchevers et al., 2021; Nelson and Bergeman, 2021; Varma et al., 2021; Bonilla et al., 2022; Leon et al., 2022), as well as our own observations of higher levels of anxiety and depression, it is plausible to hypothesize that isolation had a negative impact on emotional variables, particularly in young adults. This impact may have affected physiological or sleep structure, which could have been imperceptible subjectively but had a detrimental effect on the memory processes we evaluated.

On the other hand, changes in cognitive domains associated with normal aging are more persistent and influenced by different factors than isolation. These factors include alterations in sleep architecture, such as advanced sleep timing, longer sleep-onset latency, shorter overall sleep duration, increased sleep fragmentation, more fragile sleep, and reduced amount of slow wave sleep (Scullin and Bliwise, 2015; Mander et al., 2017; Helfrich et al., 2018). Additionally, there are structural changes in the brain, including declines in white and gray matter volume and reductions in the size and the number of connections between neurons (Harada, 2013). Therefore, we propose that a more extensive sleep hygiene intervention may be necessary to demonstrate significant positive changes in memory processes, such as encoding and memory retrieval in older adults. Another possibility is to boost the effects of brief hygiene interventions by incorporating other techniques proposed by neuroscience to improve cognition. These non-invasive techniques include reactivation of memories during sleep using odors or auditory tones, hypnosis and closed-loop acoustic stimulation, which aims to improve the quality of slow waves and positively impact consolidation and memory encoding (Feld and Diekelmann, 2015). We consider that combining the aforementioned techniques with sleep hygiene education, a simple, easy implementable, low cost without adverse effects, could yield additional benefits. In terms of combining sleep hygiene practices with, for example, acoustic closed-loop stimulation, even though these approaches are distinct from each other, research has demonstrated that acoustic closed-loop stimulation targeting slow oscillations at a frequency of 0.8 Hz can enhance the quality of these slow oscillations (Ngo et al., 2013; Papalambros et al., 2017). Moreover, implementing proper sleep hygiene practices has been associated with increased slow wave sleep, reduced micro-arousals, and decreased rebound effects caused by certain substances on the body’s sleep regulation (Chen et al., 2010; Yang et al., 2010). Thus, we hypothesize that combining these techniques may lead to a synergistic effect, resulting in favorable changes in sleep physiology and structure, as well as an improved subjective sleep experience in the medium term.

Regarding the emotional variables, this short sleep hygiene education did not impact psychological variables, such as anxiety and depression neither for younger adults nor for older adults. Previous studies used 6 to 8 weeks intervention and observed a decay in the stress level as well as an improvement on the subjective sleep quality and insomnia in young and older adults (Brown et al., 2006; Ju and Woo, 2016; Sharma and Srivastava, 2018). Thus, longer sleep hygiene interventions are required to impact on emotional variables.

Moreover, the brief sleep hygiene intervention implemented in our study could probably produce positive changes on young adults’ sleep quality, such as an increase in slow wave sleep duration and quality, resulting in memory improvements. However, these physiological changes could be imperceptible to the subject’s self-perceived sleep quality and yet positively impact cognition. In this line, the synaptic homeostasis hypothesis posits that during the slow wave sleep occurs the synapse restoration that restores cellular homeostasis and prepares us to a new round of encoding after wakening (Tononi and Cirelli, 2014). In relation to this, one limitation of our study was the inability to perform physiological measurements such as polysomnographic recordings due to the quarantine context imposed by COVID-19. Therefore, we relied solely on subjective self-reports assessed by the PSQI index. In relation to this, a review conducted by Harvey and Tang in 2012 demonstrated that individuals with insomnia often have distorted perceptions of sleep quality. They tend to overestimate the time it takes to fall asleep (sleep onset latency) while underestimating the total amount of sleep (total sleep time). These subjective perceptions of sleep quality do not align with objective measures such as polysomnography and actigraphy, which are commonly used techniques to assess sleep patterns. This perception is limited by various factors, such as the erroneous perception of sleep as wakefulness, worry and micro-arousals (Harvey and Tang, 2012). Furthermore, a study conducted by Conte et al. in 2021 examined the differences in objective sleep parameters and sleep quality between individuals who subjectively perceived their sleep as good or bad. The study evaluated two nights of total sleep in two groups: good sleepers and bad sleepers. Interestingly, the study found that classical sleep architecture parameters such as total sleep time and sleep efficiency did not differ between the two groups. However, bad sleepers showed decreased sleep continuity (frequency of awakenings), stability (frequency of awakenings and state transitions), and lower sleep organization (number of sleep cycles and time spent in cycles) (Conte et al., 2021). On the other hand, similarly to individuals with insomnia, bad sleepers experienced a dissociation between perceived sleep quality and classical sleep architecture variables, all appearing in the normal range (Harvey and Tang, 2012; Castelnovo et al., 2019). This discrepancy may be attributed to the fact that the objective factors of perceived sleep quality are based on characteristics that are not always fully accessible to conscious awareness. Considering these findings, one limitation of our study is the absence of objective sleep measures to compare with participant self-reports. We acknowledge that significant differences between groups may exist but may not have been detected due to the changes resulting from sleep hygiene interventions not being fully accessible to conscious awareness. Additionally, individual characteristics such as selective attention to sleep or distorted beliefs about sleep quality may influence the process of judging sleep quality (Harvey and Tang, 2012). Thus, subjective perception of sleep may differ from physiological measures. Despite this, our results highlight the importance of implementing simple yet effective tools like sleep hygiene, particularly in contexts of social isolation, regardless of the underlying causes. Even a short one-week intervention has demonstrated a positive impact on memory improvement in young adults, underscoring the effectiveness of sleep hygiene as a tool for enhancing memory.

## Data availability statement

The original contributions presented in the study are included in the article/Supplementary material, further inquiries can be directed to the corresponding authors.

## Ethics statement

The studies involving human participants were reviewed and approved by Alberto C. Taquini Institute Biomedical Research Ethics Committee. The patients/participants provided their written informed consent to participate in this study.

## Author contributions

LT organized the database, performed the statistical analysis and draft and wrote sections of the manuscript. CF contributed to conception and design of the study. MM, FL, RR, and LB revised the manuscript critically for important intellectual content. All authors contributed to the article and approved the submitted version.

## Funding

This work was supported by AGENCIA PICT Serie A N°02666 to CF.

## Conflict of interest

CF and RR are co-founders of Cognitio, and LT and MM are employees of Cognitio, which develop devices to improve memory cognition.

The remaining authors declare that the research was conducted in the absence of any commercial or financial relationships that could be construed as a potential conflict of interest.

## Publisher’s note

All claims expressed in this article are solely those of the authors and do not necessarily represent those of their affiliated organizations, or those of the publisher, the editors and the reviewers. Any product that may be evaluated in this article, or claim that may be made by its manufacturer, is not guaranteed or endorsed by the publisher.
