# Supplementary material for: One-week sleep hygiene education improves episodic memory in young but not in older adults during social isolation
Source: Front Psychol. 2023 Aug 1;14:1155776. doi: 10.3389/fpsyg.2023.1155776 (PMC10433204; doi:10.3389/fpsyg.2023.1155776)
Supplement: Supplementary file 1 [file Data_Sheet_1.pdf]

Supplementary material

One-week sleep hygiene education improves episodic memory in young but not in older adults during social isolation.

Short title: Sleep hygiene education improves cognition

Leonela M. Tassone[1,2*], Malen D. Moyano[1,2], Fernando Laiño[3], Luis I. Brusco[2,4], Rodrigo E. Ramele[5] & Cecilia Forcato[1*]

Correspondence:

*Corresponding Authors

ltassone@itba.edu.ar (LT)

cforcato@itba.edu.ar (CF)

Supplementary Data
S1 Data. Sleep hygiene education instructions

List of sleep hygiene activities to be carried out by the participants.

1. Maintain a routine of waking up and going to bed. Try to always wake up and go to bed at the same time.
2. Ensure a daily solar exhibition of at least 15 minutes.
3. Perform at least 20 minutes of physical activity daily. Make sure it is at least 4 hours apart from bedtime.

4. If you take a nap, the duration should be less than 30 minutes, and between 1:00 and 3:00 p.m.
5. Reduce exposure to bright light after sunset.
6. Avoid drinking coffee and alcoholic drinks in the 4 hours before going to bed.
7. If you smoke, avoid doing so in the 2 hours before going to bed.
8. Avoid the use of electronic devices (tv, cell phone) in the 2 hours before bedtime.
9. Do not take sleeping pills unless expressly indicated by a doctor, and notify the experimenter.
10. Warm hands and feet for 5 minutes at bedtime. This can be done with a water bag, or thermal pad
11. Ensure that the room to sleep is calm, without external noises or light stimulation, and with a pleasant temperature, ideally around 19 degrees.
12. Do not ruminate problems in bed.
13. If you can't sleep, get out of bed and do a quiet activity, like reading or listening to music. Go back to bed when you feel tired again.
14. Do not sleep with the television or radio on.
15. Recommended foods to eat at dinner time: pasta, milk, oatmeal, nuts, rice, chicken.
16. Foods to avoid during dinner or near sleep: fatty or spicy food, foods with high sugar content, fizzy drinks (soda).
