# Supplementary material for: One-week sleep hygiene education improves episodic memory in young but not in older adults during social isolation
Source: Front Psychol. 2023 Aug 1;14:1155776. doi: 10.3389/fpsyg.2023.1155776 (PMC10433204; doi:10.3389/fpsyg.2023.1155776)
Supplement: Supplementary file 2 [file Data_Sheet_2.pdf]

Supplementary material

One-week sleep hygiene education improves episodic memory in young but not in older adults during social isolation.

Short title: Sleep hygiene education improves cognition

Leonela M. Tassone[1*], Malen D. Moyano[1], Fernando Laiño[2], Luis I. Brusco[3,4], Rodrigo E. Ramele[5] & Cecilia Forcato[1*]

Correspondence:

*Corresponding Authors

ltassone@itba.edu.ar (LT)

cforcato@itba.edu.ar (CF)

Supplementary Data
S2 Appendix. Activities to carry out according to each participant chronotype

“Taking into consideration the different chronotypes that may exist, the recommendations are separeted in two. Take into account those that fit the chronotype that you have been informed about.

Morning and intermediate chronotype:

This routine has to be extended for every day of the week including the weekend, since this practice avoids social jetlag.

Sleep and wake-up times should be adjusted to align with the recommended schedule for your chronotype (always maintaining the wake-up and bedtime schedule, getting out of bed early in the morning, and doing activities with higher cognitive demand in the morning),

Try to ensure an approximate of 8 hours of sleep.

Social activities such as virtual meetings should be scheduled in shorter and more frequent periods throughout the week, to avoid disrupting the sleep routine.

In case of taking naps, it is recommended to take it between 1:00 p.m. and 3:00 p.m., lasting no longer than 30 minutes.
Intellectually demanding tasks must be reserved for morning or early afternoon hours, and try to avoid activities with high cognitive demands after sunset.
Physical exercise should be conducted in the morning to maintain a 6-hour difference between physical activity and bedtime.
It is recommended to allow for a sufficient period of time between the last activity of the day and bedtime to promote relaxation before going to sleep.

Evening chronotype
This routine has to be extended for every day of the week including the weekend, since this practice avoids social jetlag.
Sleep and wake-up times should be adjusted to align with the recommended schedule for your chronotype (always maintaining the wake-up and bedtime schedule, waking up late according to your biological clock, and doing activities with higher cognitive demand in the afternoon).
Try to ensure an approximate of 8 hours of sleep
It is not recommended to take afternoon naps as this can have a negative impact on nighttime sleep. This is important because late naps can impair nighttime sleep onset.
Physical exercise should be conducted in the morning or afternoon, taking care that do it at least 6 hours away from their usual bedtime.
In relation to intellectual tasks, it si recommended to do them during the afternoon.”
